# Exploiting viral sensing mediated by Toll-like receptors to design innovative vaccines

**DOI:** 10.1038/s41541-021-00391-8

**Published:** 2021-10-28

**Authors:** Rossella Sartorius, Maria Trovato, Roberta Manco, Luciana D’Apice, Piergiuseppe De Berardinis

**Affiliations:** Institute of Biochemistry and Cell Biology, C.N.R., Via Pietro Castellino 111, 80131 Naples, Italy

**Keywords:** Vaccines, Infectious diseases

## Abstract

Toll-like receptors (TLRs) are transmembrane proteins belonging to the family of pattern-recognition receptors. They function as sensors of invading pathogens through recognition of pathogen-associated molecular patterns. After their engagement by microbial ligands, TLRs trigger downstream signaling pathways that culminate into transcriptional upregulation of genes involved in immune defense. Here we provide an updated overview on members of the TLR family and we focus on their role in antiviral response. Understanding of innate sensing and signaling of viruses triggered by these receptors would provide useful knowledge to prompt the development of vaccines able to elicit effective and long-lasting immune responses. We describe the mechanisms developed by viral pathogens to escape from immune surveillance mediated by TLRs and finally discuss how TLR/virus interplay might be exploited to guide the design of innovative vaccine platforms.

## Introduction

The immune system has perfected a very organized system committed at alerting the organism from warning signs. First proposed in 1989 by Charles Janeway Jr as receptors of innate immune cells^1^, pattern-recognition receptors (PRRs, Box 1) are an old evolutionarily family of germline-encoded non-clonal proteins that function as host sensors of invading pathogens or danger signals^2–4^. Constitutively expressed in the host, PRRs are capable of detecting distinct conserved repeating patterns of molecular structures on microbes, designated as pathogen-associated molecular patterns (PAMPs), or more appropriately as microorganism-associated molecular patterns^5^. Likewise, PRRs can be engaged by endogenous molecules released upon tissue stress or damage^6^, termed damage-associated molecular patterns (DAMPs)^7^. These detection events collectively trigger downstream inflammatory pathways and antigen-specific adaptive immune responses as host defense against the invading microorganism and/or tissue damage^3,8^. Among PRRs, Toll-like receptors (TLRs) sense a wide range of infectious agents, playing a crucial role in microbial recognition and control of adaptive immunity.

Box 1 **List of abbreviations** **AAV:** adeno-associated viruses; **ACE2:** angiotensin-converting enzyme 2; **AdV:** adenoviral vector; **AP-1:** activator protein 1; **APCs:** antigen-presenting cells; **APs:** adaptor proteins; **ARDS:** acute respiratory distress syndrome; **BMDCs:** bone marrow-derived dendritic cells; **cDCs:** conventional or classical dendritic cells; **CHIKV**: Chikungunya virus; **CMV:** cytomegalovirus; **COPII:** coat protein complex II; **CoV:** coronavirus; **COVID-19**: Coronavirus Disease 2019; **CpG:** cytosine–phosphate–guanosine; **CRAMP**: cathelin-related antimicrobial peptide; **CSFV:** classical swine fever virus; **CVB3:** Coxsackievirus B3**; DAMPs:** damage-associated molecular patterns; **DCs:** dendritic cells; **DD:** death domain; **DENV:** Dengue virus; **DF:** dengue fever; **DHF:** dengue haemorrhagic fever; **dsDNA**: double-stranded DNA; **dsRNA:** double-stranded RNA; **DSS:** dengue shock syndrome; **ECs:** endothelial/epithelial cells; **ECD:** extracellular domain; **EMCV:** encephalomyocarditis virus; **Env:** envelope; **ER:** endoplasmic reticulum; **ERKs:** extracellular signal-regulated kinase kinases; **EV71:** Enterovirus 71; **GBS:** Group B *Streptococcus*; **G*-*CSF:** granulocyte colony-stimulating factor; **GLA-SE:** glucopyranosyl lipid adjuvant-stable emulsion; **GPI-mucin:** glycosylphosphatidylinositol-mucin; **gp41:** glycoprotein 41; **GXM:** glucuronoxylomannan; **HBV:** Hepatitis B virus; **HBX:** Hepatitis B virus X protein; **HCV:** Hepatitis C virus; **HIV-1:** human immunodeficiency virus type 1; **HPV:** human papilloma virus; **HSP:** heat shock protein; **HSV:** herpes simplex virus; **HTLV-1:** human T cell leukaemia virus 1; **IAV:** influenza A virus; **ICP0:** infected cell protein 0; **IFN:** interferon; **IKK:** IκB kinase; **IL:** interleukin; **IL-1R:** interleukin 1 receptor; **ILCs:** innate lymphoid cells; **IM:** inflammatory monocytes; **IP-10:** IFN-γ-induced protein 10; **IRAK:** interleukin 1 receptor-associated kinase; **IRF:** interferon-regulatory factor; **ISDR:** interferon sensitivity-determining region; **ISRE:** interferon-stimulated response element; **ISGs:** interferon-stimulated genes; **JAK:** Janus kinase; **JNK:** c-Jun N-terminal kinase; **KCs:** Kupffer cells; **KSHV:** Kaposi’s sarcoma-associated herpesvirus; **LAM:** lipoarabinomannan; **LBP:** lipopolysaccharide binding protein; **let-7 miRNA:** lethal-7 microRNA; **LPS:** lipopolysaccharide; **LRR:** leucine rich repeat; **LSECs:** liver sinusoidal endothelial cells; **LTA:** lipoteichoic acid; **MAL:** MyD88-adapter-like protein; **MAMPs:** microorganism-associated molecular patterns; **MAPK:** mitogen-activated protein kinase; **MCPs:** monocyte chemoattractant proteins; **MCs:** mast cells; **MCV:** molluscum contagiosum virus; **mDC:** myeloid DCs; **MERS:** Middle East Respiratory Sindrome; **MEFs:** mouse embryonic fibroblasts; **MIP*:*** macrophage inflammatory protein*;* **MLV:** murine leukemia virus; **MMTV:** mouse mammary tumor virus; **MPL:** monophosporil lipid; **mtDNA:** mitochondrial DNA; **MyD88:** myeloid differentiation primary-response protein 88; **MRV:** mammalian orthoreovirus; **MV:** measles virus; **MVA:** modified Vaccinia virus Ankara; **NAP1:** NF-κB-activating kinase associated protein 1; **NEMO:** NF-κB essential modulator; **NF-κB:** nuclear factor kappa-light-chain-enhancer of activated B cells; **NK:** natural killer; **NoV:** Norovirus; **NPs:** nanoparticles; **NSPs:** non-structural proteins; **ODN:** oligodeoxynucleotides; **ORN:** oligoribonucleotide; **PAMPs:** pathogen-associated molecular patterns; **PBMCs:** peripheral blood mononuclear cells; **pDCs:** plasmacytoid dendritic cells; **PEI:** polyethylenimine **PG:** peptidoglycan; **PLGA:** poly(lactic-co-glycolic) acid; **PLM:** phospholipomannan; **poly-M:** mannuronic acid polymers; **PRRs:** Pattern-Recognition Receptors; **RANTES:** Regulated upon Activation, Normal T Cell Expressed and Presumably Secreted; **RIPK1:** receptor-interacting protein kinase 1; **rRNA:** ribosomal RNA; **RSV:** respiratory syncytial virus; **RTA:** replication and transcription activator; **RV:** rhinovirus; **SFV:** Semliki Forest virus; **SARM:** sterile-α and Armadillo motif-containing protein; **SARS:** Severe Acute Respiratory Sindrome; **SARS-CoV-2:** severe acute respiratory syndrome coronavirus 2; **SINTBAD:** similar to NAP1 TBK1 adaptor; **SMOCs:** supramolecular organizing centers; **SOCS-1:** suppressor of cytokines signalling; **ssDNA:** single-stranded DNA; **ssRNA:** single-stranded RNA; **STAT:** signal transducer and activator of transcription; **TAB:** TAK1-binding protein; **TAMs:** tumor-associated macrophages; **TANK:** TRAF family member-associated NF-kappa-B activator; **TAK1:** transforming growth factor β-activated kinase 1; **TBK1:** NF-κB activator (TANK) binding kinase 1; **TgPRF:** *Toxoplasma gondii*-derived profilin-like proteins; **TICAM-1:** TIR domain-containing adaptor molecule-1; **TIR:** Toll/interleukin 1 receptor; **TIRAP:** TIR domain-containing adaptor protein; **TLRs:** Toll-like receptors; **TMD:** transmembrane domain; **TNF:** tumor necrosis factor; **TNFR1:** TNF-α receptor 1; **TRAF:** tumor necrosis factor receptor-associated factor; **TRAM:** translocating chain-associating membrane protein; **TRIF:** TIR domain-containing adaptor protein inducing interferon-β; **UPEC:** uropathogenic *E. coli*; **VACV:** Vaccinia virus; **VLPs:** virus-like particles ; **VSV:** vesicular stomatitis virus; **WNV:** West Nile virus.

## TLRs: structure, phylogenetic subfamilies, localization, and ligands

Pioneering findings on mechanisms driving the antifungal responses in *Drosophila melanogaster* were crucial to the discovery of TLRs and their function in mammalian immunity^9^. The identification in 1997 of TLR4 as the ortholog of *Drosophila* Toll protein^10^ and subsequently as the lipopolysaccharide (LPS) receptor^11^ was an event of utmost importance that stirred up a considerable interest in this field^12^. To date, 10 members (TLR1–TLR10) of TLR family have been identified in humans and 12 (TLR1–TLR9 and TLR11–TLR13) in mice, with TLR10 non-functional in the latter^13^.

**Fig. 1:**
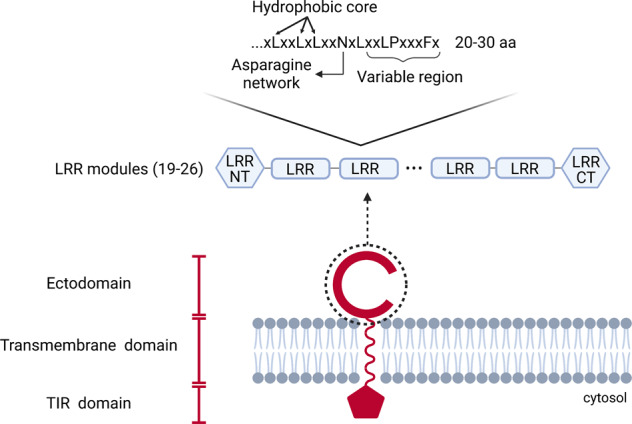
Schematic overview of the TLR structure. A TLR is composed of: (i) an extracellular ligand-binding domain (ECD) containing various numbers of LRR modules (19–26); (ii) a single transmembrane α-helix domain (TMD); and (iii) a TIR domain responsible for the downstream signaling cascade. In cases of intracellular TLRs, the N-terminal ECD is directed toward the lumen of the intracellular compartments. Each LRR module is 20–30 amino acids (aa) in length and has a consensus “LxxLxLxxNxL” sequence and a variable region. The consensus sequence, located in the concave surface of the protein, forms parallel β-strands and ligand-binding sites. It contains the hydrophobic core and the asparagine network, which ensure the protein stability. Created with BioRender.com.

All TLRs share a common structural organization: they are type-I transmembrane glycoproteins with an extracellular recognition domain, a single transmembrane helix, and a cytoplasmic signaling domain (Fig. 1).

The N-terminal ectodomain consists of varying numbers (19–26) of leucine rich repeat (LRR) modules, each of which is 20–30 amino acids in length containing the consensus “LxxLxLxxNxL” sequence motif, separated from the transmembrane region by the “LRR carboxy-terminal domain”^14^. The ectodomain displays the characteristic horseshoe-like shape and mediates recognition of PAMPs/DAMPs^4,15,16^. The intracellular domain (of ~150 amino acid residues), named Toll/interleukin 1 receptor (IL-1R) homology (TIR) domain for its similarity to that of IL-1R, is carboxy-terminal to LRRs. This domain initiates the downstream signal transduction pathways, recruiting TIR domain-containing adapter proteins (APs)^17,18^.

**Fig. 2:**
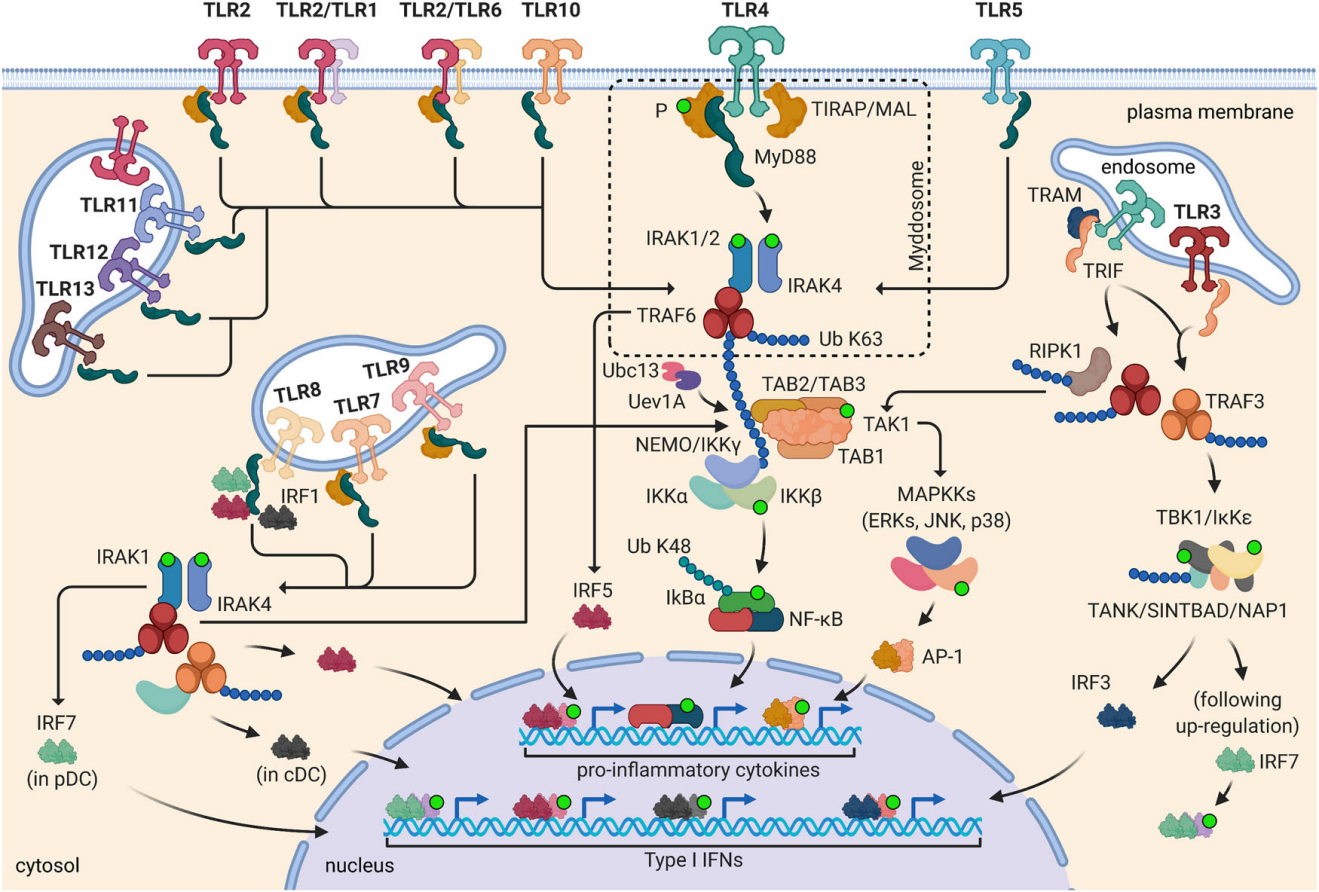
Schematic overview of TLR signal transduction. Within myddosome, the C-terminal TIR domain of MyD88 interacts with TIR domains of TLRs and TIRAP/MAL (required for TLR2, TLR4, TLR7, and TLR9 signaling), whereas the N-terminal death domain (DD) recruits by homotypic interactions IRAK4, which in turn activates IRAK1 and IRAK2. These serine/threonine kinases promote both auto-phosphorylation and trans-phosphorylation of IRAK members with subsequent recruitment and activation of TRAF6, which plays as an E3 ubiquitin protein ligase. Together with E2 ubiquitin-conjugating enzymes Ubc13/Uev1A, TRAF6 promotes polyubiquitination and activation of TAK1 complex, consisting of TAK1, TAB1, TAB2, and TAB3. This leads to phosphorylation of IKK complex, composed of IKK-α, IKK-β, IKK-γ (NEMO) subunits, and consequently of IκBα, a NF-κB inhibitory protein. Phosphorylated IκBα is degraded by the proteasome, freeing NF-κB to translocate into the nucleus and activate the expression of pro-inflammatory cytokine genes. Simultaneously, TAK1 triggers the MAPK cascades, leading to the formation and activation of AP-1 transcription factor complex. IRAK complex and TRAF6 interact also with IRF5, resulting in phosphorylation and nuclear translocation of IRF5, which binds ISRE motifs into the promoter regions of IFN-inducible cytokine genes. TLR7, TLR8, and TLR9 signaling induces also type I IFN (IFN-α and IFN-β) production in a MyD88-dependent manner through nuclear translocation of IRF7 (in pDCs), a key transcription factor for IFN-α induction, and IRF1 (in cDCs), culminating into IFN-β expression. In macrophages and cDCs, downstream of dimerized TLR3 and TLR4, the MyD88-independent/TRIF-dependent pathway leads to the expression of IFN and ISGs through recruitment of TRAM, TRIF, and TRAF3. On endosomes, the bridging factor TRAM interacts with the dimerized TLR4 and TRIF to drive TBK1 activation through TRAF3 (TRAF3–TBK1–IKKε axis), resulting in IRF3 phosphorylation and dimerization. TRIF interacts also with TRAF6 and RIPK1 to activate NF-κB and MAPKs through TAK1 complex, and consequently AP-1. Subsequent nuclear translocation of activated transcription factors IRF3, NF-κB, and AP-1 leads to expression of type I IFNs, ISGs, and pro-inflammatory cytokines. Activation of constitutively expressed IRF3 together with type I IFNs promotes activation of IRF7, through the JAK-STAT pathway, enhancing expression of type I and III IFNs. Created with BioRender.com.

TLRs localize to the cell surface and/or reside within intracellular compartments, such as endosomes, multivesicular bodies, lysosomes, and endolysosomes. Cell-surface TLRs include TLR1, TLR2, TLR4, TLR5, TLR6, and TLR10, while intracellular TLRs comprise TLR3, TLR7, TLR8, TLR9, TLR11, TLR12, and TLR13^19^ (Fig. 2). TLR2 and TLR4 are also expressed intracellularly in dendritic cells (DCs), epithelial cells (ECs), and endothelial cells^20^. Based on their amino acid sequences, TLRs can be further divided into six major phylogenetic subfamilies, sensing related PAMPs: the TLR1, TLR3, TLR4, TLR5, TLR7, and TLR11 subfamilies^21^. In mammals, TLRs are synthesized in the endoplasmic reticulum (ER) where the ER-resident chaperone proteins gp96 (also known as Grp94), PRAT4A (also known as Cnpy3), and UNC93B1 support them for proper folding and efficient translocation^3^. While cell-surface TLRs likely traffic from ER to plasma membrane through the Golgi complex via the conventional secretory pathway, the intracellular trafficking of TLRs to endosomes is regulated by UNC93B1 and specific trafficking APs. UNC93B1 promotes TLR incorporation into coat protein complex II vesicles, budding off the ER, to subsequently cross the Golgi complex. Likely, AP-4 mediates trafficking of TLR7, TLR11, TLR12, and TLR13 directly from the Golgi to endosomes. In contrast, TLR9 is delivered to the plasma membrane and then into endolysosomal compartments via AP-2-mediated endocytosis. Within endosomes, cathepsins and proteases promote cleavage of TLR ectodomains, a prerequisite for signal transduction^3,22^.

TLRs detect a wide spectrum of microbial components, host-derived ligands (Table 1), or synthetic compounds (detailed afterwards).

**Table 1:** Subcellular localization and natural ligands of TLRs.

TLR	Localization	Ligands	Origin of ligands	References
TLR2/TLR1	Plasma membrane	Diacyl/triacyl lipopeptides	Bacteria/mycobacteria	^2,16,31^
TLR2	Plasma membrane/endosome	Lipoproteins PG *Neisseria* porins *C. albicans* PLM *C. neoformans* GXM *S. cerevisiae* zymosan LAM *Trypanosoma* GPI-mucin Envelope glycoproteins	Multiple pathogens Bacteria Bacteria Fungi Fungi Fungi Mycobacteria Protozoa Viruses	^2,16,31,45^
TLR2/TLR6	Plasma membrane	Diacyl lipopeptides GBS LTA *S. cerevisiae* zymosan Envelope glycoproteins	Bacteria/mycobacteria Bacteria Fungi Viruses	^2,16,31^
TLR3	Endosome	dsRNA	Viruses/bacteria/endogenous	^31–33^
TLR4	Plasma membrane/endosome	LPS *P. aeruginosa* poly-M *C. albicans* mannan *C. neoformans* GXM HSP60 and HSP70 Fibrinogen Envelope glycoproteins Nickel/cobalt/platinum	Bacteria Bacteria Fungi Fungi Endogenous Endogenous Viruses Metals	^18,34,69,167^
TLR5	Plasma membrane	Flagellin	Bacteria	^168^
TLR7	Endosome	ssRNA let-7 miRNA	Viruses/bacteria Endogenous	^31,32,169,170^
TLR8	Endosome	ssRNA	Viruses/bacteria/archaea	^31,32,169,170^
TLR9	Endosome	ssDNA mtDNA	Bacteria/protozoa/viruses Endogenous	^59^
TLR10	Plasma membrane	HIV-1 gp41 *H. pylori* LPS (TLR2/10) *L. monocytogenes* *B burgdorferi* H1N1/H5N1	Viruses Bacteria Bacteria Bacteria viruses	^45^
TLR11	Endosome	*Salmonella*/*E. coli* flagellin UPEC TgPRF	Bacteria Bacteria Protozoa	^171,172^
TLR12	Endosome	TgPRF	Protozoa	^173^
TLR13	Endosome	23S rRNA VSV ssRNA	Bacteria Viruses	^57,58^

Interaction with ligands (or agonists) induces homodimerization or heterodimerization of TLR ectodomains, with subsequent dimerization of their intracellular domains, recruitment of signaling adapters, and activation^14,15,18^. Cell-surface TLRs mainly bind to proteins, lipids, and lipoproteins (TLR1, etc.), while intracellular TLRs detect nucleic acids (NAs; TLR3, TLR7, TLR8, TLR9, TLR13) or microbial components (TLR11, TLR12) derived from endolysosomal degradation.

## TLR signal transduction

TLRs are expressed in many cell types (reviewed in ref. ^20^). These include either non-immune cells, such as endothelial cells/ECs, or innate immune cells like monocytes/macrophages, mast cells (MCs), neutrophils, eosinophils, basophils, natural killer (NK) cells, γδ T cells, innate lymphoid cells, DCs, platelets; brain innate immune cells like microglia and astrocytes; and cells of adaptive immunity: T and B cells. Distinct cell types show differential patterns of TLR expression, depending on cell subtype, activation status, developmental stage, and species^20,23–25^.

TLR engagement by ligands induces conformational changes required to recruit downstream adapter molecules to dimerized receptor TIR domains for triggering distinct signaling pathways that culminate into transcriptional upregulation of different genes involved in immune defense^26^. In humans, TLR signaling activation involves five APs: MyD88 (myeloid differentiation primary-response protein 88); MAL (MyD88-adapter-like protein) also known as TIRAP (TIR domain-containing AP); TRIF (TIR domain-containing AP inducing interferon-β) also termed TICAM-1 (TIR domain-containing adapter molecule-1); TRAM (translocating chain-associated membrane protein) also named TICAM-2; and SARM (sterile-α and Armadillo motif-containing protein)^18^. MyD88 is the key adapter for all TLRs. However, TLR3 signals exclusively via TRIF, while TLR4 (unique among TLRs) initiates a MyD88-independent/TRIF-dependent pathway upon translocation from the plasma membrane to endosomes in a CD14-dependent manner^27^. SARM negatively regulates TRIF and thus controls signaling of TLR3 and TLR4^28^.

Upon ligand binding of dimerized TLR, TIR domains are recognized by TIRAP/MAL and TRAM bridging factors. These factors induce the assembly of supramolecular organizing centers (SMOCs) around the cytosolic tail of dimerized TLRs. All TLRs engage a SMOC namely “myddosome” seeded by TIRAP/MAL, which leads to the induction of pro-inflammatory cytokines (MyD88-dependent pathway) that promote the synthesis of additional inflammatory mediators. Conversely, TLR3 and TLR4 engage a SMOC likely triggered by TRAM on endosomes, stimulating the production of type I interferons (IFNs) (MyD88-independent/TRIF-dependent pathway) (Fig. 2) that mediate antiviral responses^3^. Collectively, these events trigger inflammation and host defense, resulting in killing of microbes/pathogens.

## TLR response to viruses

Recognition of viral PAMPs by TLR ectodomain triggers intracellular downstream signaling pathways that culminate in the antiviral immune responses^29^. IFN signaling cascade plays a key role in control of viral infection, activating the Janus kinase/signal transducer and activator of transcription (STAT) pathway and upregulating transcription of antiviral IFN-stimulated genes (ISGs). Through their own paracrine/autocrine activity, the IFN/ISG system blocks the viral multiplication in the infected cells and maintains an antiviral state in uninfected neighboring cells by preventing viral entry, replication, and budding^30^. In addition, inflammatory cytokines regulate the maturation of innate and adaptive immune cells and control their recruitment to the site of infection. Subsequently, the activated adaptive immune responses lead to viral clearance by neutralizing antibodies and cytotoxic CD8^+^ T cells^31^. In the following sections, we have outlined the TLR response to viruses upon detection of viral double-stranded RNA (dsRNA), single-stranded RNA (ssRNA), double-stranded DNA (dsDNA), and proteins.

### TLR recognition of viral dsRNA

#### Role of TLR3

TLR3 detects dsRNA, including synthetic dsRNA analogs (e.g., poly I:C), genome of dsRNA viruses, phagocytosed host RNA, or intermediates generated during viral replication of ssRNA viruses^31–33^. During the life cycle of viruses, the viral genetic material may alert intracellular TLRs. Among them, TLR3 senses viral dsRNA in endosomes as pathogenic non-self and responds by increasing its expression, promoting IFN-β responses and release of inflammatory cytokines as host defense against the viral invasion^34^. Mammalian orthoreovirus (MRV), a member of the Reoviridae family, was the first segmented dsRNA virus identified in the 1950s. Among the four serotypes, MRV Type I Lang can be sensed by TLR3. Detection of viral dsRNA induces IFN-β, IL-12, IL-6 and tumor necrosis factor-alpha (TNF-α) expression. IFNs inhibit viral replication, activating transcription of proteins responsible for RNA degradation, inhibitors of translation of viral mRNAs, and modulators of antigen processing and presentation; IL-6 and IL-12 cytokines mediate active cytotoxic responses^35^.

In addition, TLR3 senses dsRNA intermediates generated during the life cycle of ssRNA viruses. Rhinovirus (RV), a ssRNA virus of Picornaviridae family, is frequently associated with human respiratory diseases, being the major cause of common cold. RV replication upregulates TLR3 mRNA and increases surface expression of this receptor, leading to an enhanced production of pro-inflammatory mediators, such as IL-6, C-X-C chemokine motif ligand 8 (CXCL8 or IL-8), and C-C chemokine motif ligand 5 (CCL5), that recruit and activate cells against the viral invasion^36^.

Hepatitis C virus (HCV) is a ssRNA virus of the Flaviviridae family. Persistent HCV infections are associated with progressive liver fibrosis and hepatocellular carcinoma. Similarly to RV, HCV is sensed by TLR3 through detection of dsRNA intermediates in infected hepatoma cells. The activated TLR3 signaling cascade leads to the synthesis of type I and II IFNs, expression of ISGs, and pro-inflammatory cytokines that limit HCV replication. Nevertheless, treatment with IFNs eliminates the virus only in 50% of HCV-positive patients. Indeed, the virus has evolved a number of strategies to antagonize the TLR3 signaling cascade (described below) and disruption of the TLR3–TRIF axis seems to determine the outcome of the infection^37^.

Dengue virus (DENV), a ssRNA virus belonging to the Flaviviridae family, causes diseases as dengue fever, dengue hemorrhagic fever, and dengue shock syndrome, whose severity can depend on persisting high viral load and high inflammatory cytokine levels in the plasma. There are four DENV serotypes (DENV-1–4). At the early stage of DENV-2 infection, TLR3 activation triggers in vitro IL-8, IL-6, IFN‐α, and IFN-β that regulate the suppression of viral replication and reduce the cytopathic effect of DENV-2^38^.

Chikungunya virus (CHIKV) is a ssRNA virus of the Togaviridae family. In response to CHIKV infection, TLR3 plays a key role in mediating neutralizing antibody responses against the virus^39^.

TLR3-dependent responses might result in detrimental disease outcomes depending on the type of virus, as the case of West Nile virus (WNV), a ssRNA of Flaviviridae family, or influenza A virus (IAV), a ssRNA of Orthomyxoviridae family. Upon detecting WNV, TLR3-dependent inflammatory responses compromise TNF-α receptor 1 signaling, crucial for blood–brain barrier, facilitating the viral entry into the brains of mice with subsequent lethal encephalitis^40^. IAV is clinically the most important cause of acute pneumonia, associated with synthesis of inflammatory cytokines and chemokines, including IL-6, granulocyte colony-stimulating factor, IL-12p40/p70, monocyte chemoattractant proteins, macrophage inflammatory proteins, and RANTES (Regulated upon Activation, Normal T Cell Expressed and Presumably Secreted). IAV-infected TLR3^−/−^ mice had an unexpected advantage to IAV infection, with reduced levels of inflammatory mediators, decreased CD8^+^ T cell infiltration, and prolonged survival, likely due to a dysregulated TLR3-dependent CD8^+^ T cell response that may lead to a sustained lung injury^41^.

dsRNA may also be generated by viruses harboring a dsDNA genome as Vaccinia virus (VACV), the prototype member of the Poxviridae family. The activated TLR3 pathway promotes production of cathelin-related antimicrobial peptide that blocks VACV replication. Unexpectedly, deletion of TLR3 in mice decreased viral replication and reduced manifestations of infection, suggesting a detrimental effect of the interaction between TLR3 and VACV^42^.

Similarly to VACV, Herpes simplex virus (HSV) that harbors a dsDNA genome is detected by TLR3 through dsRNA replication intermediates. The immune response to HSV-1 can depend on viral subcellular localization during intracranial infection with the virus, relying on the TLR3–mTORC2–mTORC1 axis. In perinuclear Rab7a+ lysosomes of infected neurons and astrocytes, the active TLR3, forming a complex with mTORC2 and TRAF3, allows chemokine production and TLR3 trafficking to the cell periphery. Peripheral TLR3 interacts with type I IFN signaling molecules, such as TRAF3 and mTORC1, leading to the production of IFN-β^43^.

Additionally, in DCs TLR3 detects phagocyted cell-associated viral dsRNA, promoting cross-priming of cytotoxic T cells against virally infected cells as demonstrated for encephalomyocarditis virus and Semliki Forest virus^44^.

#### Role of TLR10

Closely related to TLR1 and TLR6, TLR10 can form both homodimers and heterodimers with TLR1, TLR2, and TLR6, although the biological function of TLRs in complexes still remains to be elucidated^45^. TLR10 binds dsRNA in endosomal compartments and negatively regulates the type I IFN response by reducing phosphorylation of IRF7. Moreover, in vitro overexpression of TLR10 subtracts dsRNA from TLR3 binding and enhances expression of SARM1, a negative regulator of TLR3 signaling pathway^46^.

### TLR recognition of viral ssRNA

#### Role of TLR7/TLR8

The highly homologous TLR7 and TLR8 are ssRNA sentinels. Both TLRs have two ligand-binding sites: one for nucleosides, which are guanosine (G) and uridine (U) for TLR7 and TLR8, respectively; the other for U- or UG-harboring short oligonucleotides, for TLR7 and TLR8, respectively^47,48^. TLR7 and TLR8 mainly detect endocytosed viruses with ssRNA genome and both activate synthesis of IFNs and pro-inflammatory cytokines. In mice, TLR7 function is well defined, while TLR8 might be not functional. TLR7 activation in plasmacytoid DCs (pDCs) leads to cross-priming promotion and induction of high levels of type I IFNs, regulating type I T helper (Th1) responses and B cells isotype switching, as it occurs upon activation with IAV or synthetic oligoribonucleotides mimicking human immunodeficiency virus (HIV) genomic sequence^49^. HIV-1 RNA is essential for pDC activation, primarily via TLR7, and production of IFN-α that upregulates the activation marker CD38 on CD8^+^ T cells^50^.

In pDCs, binding of ssRNA viruses to TLR7 induces INF-α and IL-12 secretion. The first step necessary to trigger TLR7-dependent responses is the acidification of the endosomal compartments^51^. Indeed, inhibitors of lysosomal acidification reduce INF-α and IL-12 production by pDCs following vesicular stomatitis virus (VSV), IAV, or Sendai virus infection^51^.

An IFN-independent antiviral response of TLR7 was also demonstrated in mouse model studies of WNV encephalitis. TLR7 could detect WNV ssRNA immediately after viral entry before recognition of dsRNA intermediates by TLR3. It has been hypothesized that resident macrophages secreting IL-23 when TLR7 detects the virus, promote infiltration and homing of peripheral immune cells, and regulate the viral neutralization and clearance^52^.

Emerging evidences suggested an important role of TLR7 during Enterovirus 71 (EV71) infection, where overexpression of pro-inflammatory cytokines plays an important role in disease severity. EV71 ssRNA is recognized by TLR7 and TLR8 in ECs, with subsequent enhancement of IFN-β^53^. Upregulation of TLR7, induction of IL-6, and apoptosis of astrocytic cells are triggered in the human brain upon EV71 infection^54^.

Interestingly, TLR8 can affect antiviral responses and expression of TLR7. In TLR8-knockout mice, TLR7 expression is upregulated and a lupus-like autoimmunity develops due to an increased TLR7 function^55^. An increased expression of type I IFN genes is observed in TLR8^−/−^ mice, while in wild-type mice TLR8 facilitates WNV infection as a result of downregulation of type I IFN-dependent antiviral responses. Following WNV infection, the expression of SOCS-1 (suppressor of cytokines signaling) is induced that inhibits STAT1-dependent IFN-α signaling, regulating aberrant inflammation in the brain. SOCS-1 expression is significantly reduced in brain tissue and neurons of TLR8^−/−^ mice following WNV infection, suggesting that TLR8 can interact with SOCS-1 to inhibit the expression of TLR7 and the molecules involved in the antiviral signaling, facilitating WNV infection^56^.

#### Role of TLR13 in mouse

Given its intracellular localization, TLR13 could bind NAs. Accordingly, murine TLR13 is engaged by bacterial 23S ribosomal RNA and was shown to be able to recognize VSV ssRNA genome, through a similar RNA motif containing a highly conserved 13-nucleotide sequence^57,58^, activating IRF7, but not IRF3, with subsequent IFN-β production. Silencing of TLR13 in VSV-infected mouse embryonic fibroblasts results in a high viral titer relative to untreated cells, indicating a TLR13-mediated response against VSV^58^.

### TLR recognition of viral ssDNA

#### Role of TLR9

TLR9 is the only known endosomal ssDNA sensor. This receptor detects the unmethylated DNA with cytosine–phosphate–guanosine (CpG) motifs derived from bacteria and viruses^59^. DNA viruses are detected by TLR9 within endosomes through the unmethylated CpG sequences. Localization of TLR9 in endosomal vesicles and endosomal acidification allow activation of immune responses against pathogen-derived DNA. Ligation of TLR9 leads to production of IFNs or pro-inflammatory cytokines as TNF-α, IL-6, and IL-12. TLR9 along with TLR7 is highly expressed in pDCs^34^. It has been suggested that TLR9 might cooperate with TLR7 in recognizing NAs from murine cytomegalovirus (CMV), a dsDNA virus of the β-herpesviridae subfamily, widely used as a model for human CMV infection. In vitro, IL-12p70 and IFN-α/β production from murine CMV infected pDCs is strictly dependent on TLR9, while TLR7 mediates IFN-α/β and TNF-α production^60^.

Upon HSV recognition, TLR9 triggers IFN-α and inflammatory cytokine production from pDCs. In the brains of mice infected by HSV, induction of TNF-α and CXCL9 are dependent on TLR9. Low levels of TNF-α and CXCL9 have been observed in TLR2^−/−^ mice and no detectable levels in TLR9^−/−^ and TLR2^−/−^/TLR9^−/−^ mice, likely due to decreased type I IFN production. Consequently, these mouse models show higher viral load in the brain and rapid development of symptoms of HSV infection. A recent study demonstrated that TLR2 and TLR9 synergistically activate multiple innate defense mechanisms that together mount an early host defense against HSV^61^.

TLR9 also detects mitochondrial DNA containing unmethylated CpG, released from DENV-infected human DCs, inducing production of type I IFNs^62^. TLR9 is also activated by DAMPs released from heart cells infected by Coxsackievirus B3, an enterovirus implicated in viral myocarditis. With a feedback mechanism, released DAMPs trigger inflammatory responses (including TNF-α and IL-6) that in turn induce further release of DAMPs, due to the damage of other cardiomyocytes, overall causing a severe myocarditis. Mice lacking TLR9 have partial recovery of the heart function and reduced cardiac inflammation^63^.

### TLR recognition of viral proteins

Surface TLRs, such as TLR2, TLR4, and TLR10, can trigger antiviral immune responses by detecting the virus coat proteins.

#### Role of TLR2

TLR2 detects several components from bacteria, fungi, parasites, and viruses, cooperating also with TLR1 and TLR6. Mycoplasmal diacylated lipopeptides can activate both TLR2/TLR1 and TLR2/TLR6 heterodimers, while bacterial triacylated lipopeptides are detected by the TLR2/TLR1 complex. Additionally, TLR2 senses viral invasion through detection of virus coat proteins and glycoproteins^2,16,31^. TLR2 detects the glycoprotein B and H from CMVs, mediating nuclear factor kappa-light-chain enhancer of activated B cells (NF-κB) activation and induction of pro-inflammatory cytokines^64^. In the spleens of murine CMV-infected mice, viral clearance is dependent on NK cell activity, TLR2-dependent IFN-α/IFN-β, and IL-18 production, which can influence NK cell proliferation^65^. Although the signaling mechanism is unclear, specialized cells called “inflammatory” monocytes activate TLR2, which leads to the production of type I IFNs, early blocking the viral replication^66^.

TLR2/TLR6 heterodimers regulate the innate response against respiratory syncytial virus (RSV), triggering TNF-α, IL-6, CCL2, and RANTES production, crucial for suppressing the viral replication in mice. Furthermore, the rapid production of CCL2, a potent leukocyte chemoattractant, promotes migration of neutrophils and activation of DCs within the lung^67^.

Measles virus also activates TLR2 by hemagglutinin, inducing pro-inflammatory cytokines such as IL-6 in human monocytic cells and the surface expression of the host target receptor CD150^68^.

#### Role of TLR4

The canonical TLR4 ligand is LPS, the major component of the outer membrane of Gram-negative bacteria. After binding in the bloodstream through the LPS-binding protein, LPS is transferred to CD14, a glycosylphosphatidylinositol-anchored membrane protein, and then to MD-2 protein, which associates with TLR4 to form the functional LPS receptor^18^. Additionally, TLR4 detects several viral glycoproteins including the fusion (F) protein from RSV and the Envelope (Env) protein from mouse mammary tumor virus (MMTV)^2,69^. TLR4 recognizes RSV through the F protein, inducing IL-6 production^70^. A protective role of TLR4 has been demonstrated in several other viral infections such as Kaposi’s sarcoma associated herpesvirus (KSHV) or VACV, but several studies confirm that TLR4 can also promote viral expansion, as in the case of MMVT infection. MMVT is a breast milk-acquired virus that first infects B cells in Peyer’s patches of the gut. MMVT Env proteins can bind TLR4 and induce maturation of bone marrow-derived dendritic cells (BMDCs), increasing the levels of costimulatory molecules, TNF-α, IL-6, and IL-12p40. Conversely, interaction of MMTV with DCs via TLR4 would favor the infection by increasing the expression of the viral cell entry receptor CD71^71^.

#### Role of TLR10

The innate response mediated by TLR10 is still not well defined: both anti-inflammatory and inflammatory functions have been reported in independent studies^45^. Recently, a role of TLR10 in sensing HIV-1 has been described. In detail, glycoprotein 41 (gp41) has been identified as the ligand responsible for the increased expression of TLR10, IκBα activation, and IL-8 production during HIV-1 infection^72^.

TLR10 induces pro-inflammatory responses also upon IAV infection, even though the viral component has not yet been defined. TLR10 expression is enhanced in a paracrine manner in uninfected cells, probably mediated by TNF-α released by human macrophages upon viral infection. It has been reported that also IL-8, IL-6, IFN-β, and IL-29 secretion depends on TLR10, since silencing of this receptor suppresses their mRNA transcription^73^.

### Sensing of SARS-CoV-2

The onset of the recent coronavirus disease 2019 (COVID-19) pandemic has put the spotlight on the cellular sensing mechanisms of human Betacoronaviruses (β-CoVs). β-CoVs typically infect the upper respiratory tract and are associated with relatively mild respiratory diseases, including common cold, fever, dyspnea, or with severe respiratory diseases, as the Severe Acute Respiratory Syndrome (SARS) and the Middle East Respiratory Syndrome (MERS). Among β-CoVs, SARS-CoV-2 (Severe Acute Respiratory Syndrome Coronavirus 2), the causative agent of COVID-19, causes illness with a wide spectrum of symptomatology, ranging from asymptomatic infection to severe acute interstitial pneumonia, alveolar damage, and acute respiratory distress syndrome (ARDS)^74^. These clinical manifestations are frequently subsequent to the development of a “cytokine release syndrome” or “cytokine storm,” that is a dysregulated overproduction of circulating pro-inflammatory cytokines, including IL-1, IL-6, IL-10, TNF-α, TNF-β, and IFNs^75^.

TLRs have been recently proposed as one of the host sensors of SARS-CoV-2. Indeed, circulating levels of DAMPs (including beta-defensin-3, high-mobility group box-1, fibrinogen, heat shock protein 70, and syndecan) activating the TLR pathways have been found in COVID-19 patients^76^. SARS-CoV-2, as the other β-CoVs, harbors a ssRNA genome, so it is reasonable to suppose that the virus is detected by the endosomal TLR7/8, although it has not been directly demonstrated. Nevertheless, bioinformatic scanning analysis identified ssRNA fragments recognized by TLR7/8 in SARS-CoV-2, SARS-CoV, and MERS-CoV genomes, suggesting that β-CoVs can be recognized by these receptors and overall activate innate immune responses through the TLR7/8 cascades^77^. The presence of loss-of-function variants of X-chromosomal TLR7 in some young men has been recently described to correlate with a worse progression of COVID-19^78^. Recently, two ssRNA motifs present within the SARS-CoV-2 genome have been shown to activate in vivo DCs via engagement of TLR7/8 and the MyD88-dependent pathway, with sustained release of type I IFNs and pro-inflammatory cytokines, mainly leading to pulmonary inflammation^79^.

In silico analyses showed that TLR3, TLR7, TLR8, and TLR9 possess a strong binding affinity toward SARS‐CoV‐2 mRNAs encoding, respectively, the non-structural protein (NSP)-10, the Env protein, NSP8, and the Spike (S) S2 domain, indicating these proteins as putative ligands^80^. Indeed, the TLR3-mediated signaling is involved in the innate antiviral response to many RNA viruses^81^, and many evidences of a protective role of the TLR3 pathway were collected in vivo in a mouse model of infection with the mouse-adapted SARS-CoV MA15 strain^82,83^.

In addition to NA sensors, other TLRs appear to be involved in the innate immune responses to SARS-CoV-2. Molecular docking studies have proven the interaction of SARS‐CoV‐2 S protein with the extracellular domains of TLR1, TLR4, and TLR6, with the strongest binding between TLR4 and the S1 subunit of the S protein^80^. Interestingly, TLR4 and TLR4 signaling pathway proteins are shown to be upregulated in peripheral blood mononuclear cells (PBMCs) isolated from COVID-19 patients compared to those from healthy controls^84^. Putative ligands for TLR4 are the oligomannose and glycan structures present on the S1 surface. Upon binding, viral particles might be internalized through an alternative mechanism to the one mediated by the angiotensin-converting enzyme 2 (ACE2) receptor^76^. One of the proposed models suggests that, once inside the cells, the viral particles can increase the expression of ACE2 via IFNs and ISGs and/or activate MyD88-dependent pro-inflammatory pathways, rather than the TRIF/TRAM-dependent pathway, inducing acute inflammatory signaling, and thus promoting ARDS and inflammation^85^. Therefore, blocking the S-TLR4 interaction by means of TLR4 antagonists or signaling inhibitors could represent a potential way to prevent SARS-CoV-2 infection^85,86^. More recently, TLR2 has been shown acting as a sensor of the E protein of β-coronaviruses, including SARS-CoV-2, and TLR2-dependent inflammation leading to lung damage has been described. The inhibition of the TLR2 signaling was able to protect against SARS-CoV-2 symptoms in vivo, providing evidence of the role played by this innate immunity receptor in viral pathogenesis^87^.

## Mechanisms of viral evasion

**Fig. 3:**
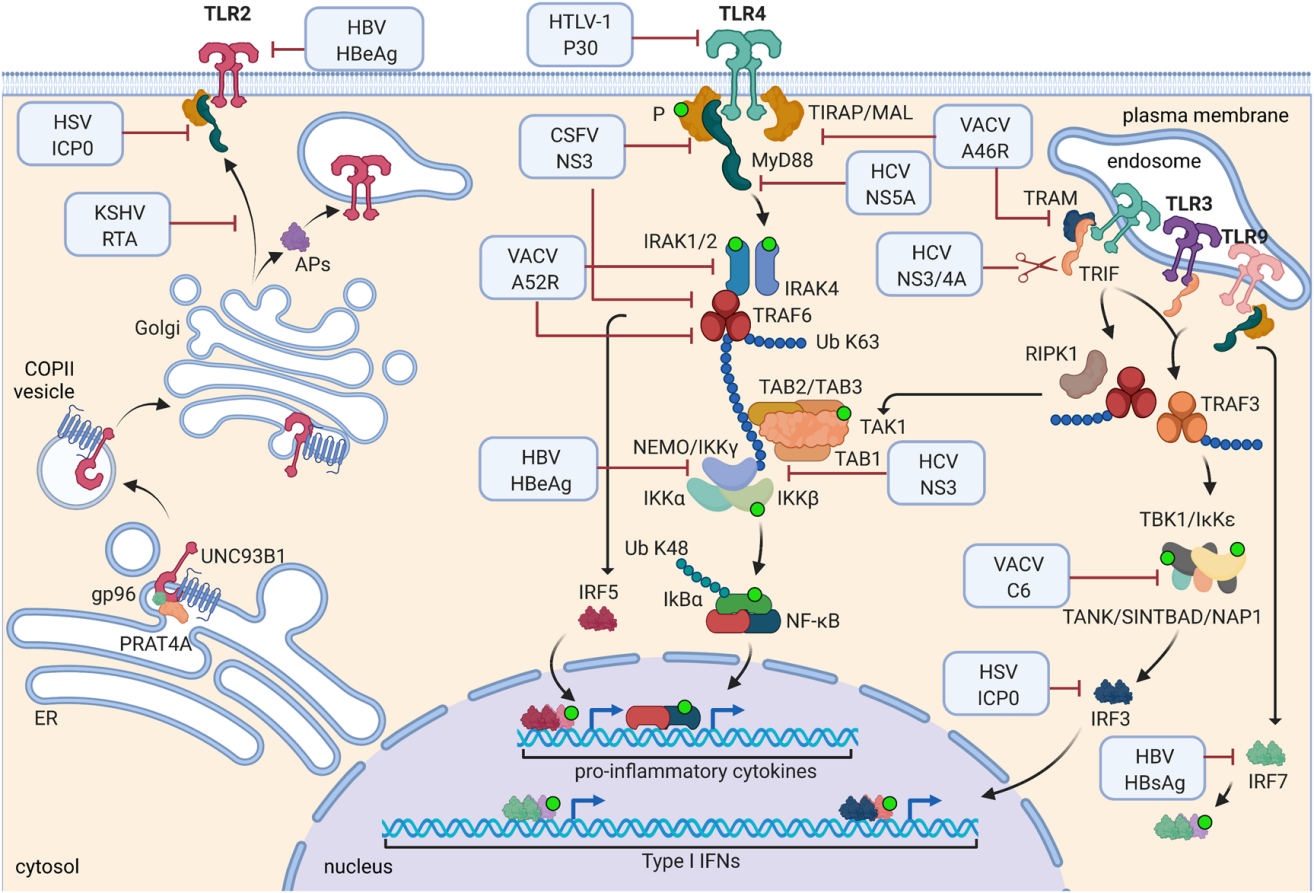
Mechanisms of viral escape from TLR sensing. Viral proteins can interfere with TLR-mediated pathways through different mechanisms. Some viral proteins, like HTLV-1 protein 30 or HBV HBeAg, directly reduce the expression of TLRs by interacting with their transcriptional factors, while KSHV RTA inhibits TLR2 and/or TLR4 localization at plasma membrane. Other viral proteins possess TIR-like domain that can bind the TIR motifs of the adapter proteins, blocking NF-κB mediated cytokine production. VACV A46R binds and sequesters MyD88, MAL, TRIF, and TRAM, while A52R interacts with IRAK2 and TRAF6, inhibiting the downstream pathway. TRAF6 can also be targeted by CSFV NSP3. Instead, VACV C6 blocks IRF3- and IRF7-mediated pathways, binding TANK, SINTBAD, and NAP1. HCV exploits its NS3/4A protease to cleave TRIF, while NS5A directly binds to MyD88, blocking its functions. Moreover, HSV ICP0 inhibits IRF3 nuclear accumulation, with subsequent impairment of IFN production. HBV HBsAg inhibits the expression and nuclear localization of IRF7, while HBeAg regulates NF-κB activity by interacting with NEMO, a strategy employed also by HCV by means of NS3. Created with BioRender.com.

In the complex interaction between host and pathogen, viruses accomplish their mission of genome widespread diffusion by transforming host cells in a bioreactor at their service. Host response is focused on the activation of the antiviral mechanisms, with most of them leading to NA-sensing selection of self versus non-self^88^. In a constant effort to escape the host defense, viruses have developed several mechanisms to counteract the host control system (summarized in Fig. 3).

Among the several mechanisms developed, interference with TLR expression is one of the best examples of host–pathogen coevolution. The strategy used by human T cell leukemia virus -1 resides on the ability of protein 30 to bind the transcription factor PU.1, regulator of TLR4 expression. PU.1 is a transcription factor expressed by B cells and macrophages. PU.1-p30 binding downregulates TLR4 expression, interfering with DC maturation and host innate immune responses^89^. Moreover, KSHV replication and transcription activator (RTA) inhibits the TLR2 and TLR4 membrane localization^90^.

Another viral strategy of immune escape is based on mirroring the host protein structure in order to mix up the immune response, as in the case of VACV. The viral A46R protein contains a TIR domain able to bind the APs MyD88, MAL, TRIF, and TRAM, and then to inhibit the downstream NF-κB activation^91,92^. The inhibition of downstream TLR signaling pathways is the strategy adopted by a large number of viruses. For instance, VACV A52R protein interacts with IRAK2 and TRAF6, inhibiting NF-κB activation^93^, while VACV C6 protein interacts with TANK, SINTBAD, and NAP1 APs, blocking IRF3 and IRF7 activation^94^.

HCV exploits several NSPs in order to interfere with TLR signal transduction: the serine protease NS3/4A, whose main function is the cleavage of viral polypeptide precursors, cleaves TRIF, inhibiting its interaction with IFN-β promoters, then cutting the antiviral signal transduction pathway started from TLRs^95^. HCV NS5A protein binds MyD88 through its IFN sensitivity-determining region, impairing the activation of TLR-mediated cytokine production^96^.

The KSHV RTA, the HSV-1 nuclear infected cell protein 0 (ICP0), the classical swine fever virus NS3 protein, the hepatitis B virus (HBV) X protein, and the CoV helper protein open reading frame-9b are examples of proteins able to block the TLR-mediated signaling, promoting degradation of downstream TLR proteins (such as MyD88, TIRAP, TRAF6)^97^. HSV-1 ICP0 protein also inhibits nuclear accumulation of IRF3 by sequestering it and accelerating its degradation^98^.

The HBV immune-evasion strategies represent a case study because the mechanisms of immune escape can drive the chronic infection. When PBMCs are cultured in the presence of TLR agonists and HBV-containing serum, TLR3-mediated IFN-γ production is inhibited; in the presence of HBeAg and HBsAg, Kupffer cells and liver sinusoidal endothelial cells, activated with TLR3 agonists, show reduced pro-inflammatory cytokine production, while anti-inflammatory cytokines are detected^99^. It has been demonstrated that the viral polymerase downmodulates INF-β production via IRF3 phosphorylation inhibition^100^. Moreover, HBsAg specifically suppressed TLR9-mediated IFN-α production by inhibiting the expression and nuclear translocation of IRF7^101^. The *e* antigen is an auxiliary viral protein, but it plays a central role in viral immune escape. Using its TIR-like domain, this protein can disrupt the homotypic TIR–TIR interaction among molecules involved in NF-κB-mediated transcription regulation and suppress TLR2- and IL-1b-mediated NF-κB activation^102^. NEMO is a regulatory subunit of IkB kinase and it is essential in controlling NF-κB activation. It has been demonstrated that HBeAg can bind NEMO impairing IL-1b-mediated NF-κB activation^103^. Several other viral proteins target NEMO, among them, the MC005 protein of molluscum contagiosum virus^104^, the HCV NS3, the VACV protein C4, and many others were described in detail elsewhere^97^.

## Exploiting TLR/virus interplay for engineering vaccine formulations

Due to their role in microbial recognition and control of adaptive immunity, several TLR agonists have been designed and exploited as adjuvants in vaccine formulations. In general, TLR agonists are well tolerated as they do not give rise to side effects, while producing dose-sparing effects, through enhancement of the vaccine efficacy. TLR4 and TLR9 agonists are the most successful and have been licensed for clinical use; agonists directed against TLR3, 5, and 7/8 need further improvements. Table 2 summarizes some of the TLR agonists used as vaccine adjuvants.

**Table 2:** TLR agonists and their application as vaccine adjuvants or therapeutic agents.

Agonist	Target	Derivative	Function	Application	Reference
Pam_3_CSK_4_	TLR1/TLR2		B cell response; T cell stimulation	Vaccine adjuvant against infections	^174^
MALP2	TLR2/TLR6		B/T cell response; pro-inflammatory cytokine production	Vaccine adjuvant against infections	^175^
Poly I:C ORN	TLR3		Type I IFN production; mDC maturation	Cancer therapeutic	^176^
		PolyI:Poly C12U	DC maturation; Th1 polarization	Cancer therapeutic	^177^
		poly-ICLC	IL-12, TNF-α, IFN-γ, IL-6, and type I IFN production	Cancer therapeutic; vaccine adjuvant against SARS-CoV	^83,178^
Monophosporil lipid (MPL)	TLR4	AS01 MPL in combination with liposome and saponin	CD4^+^ mediated immune responses	Vaccine adjuvant against Herpes zoster (Shingrix)	^179,180^
		AS04 MPL adsorbed on aluminum salts	Prolonged cytokine response at the injection site	Vaccine adjuvant against HPV (Cervarix)	^181^
		GLA-SE (glucopyranosyl lipid adjuvant-stable emulsion)	High antibody titer induction	Vaccine adjuvant against influenza virus	^182,183^
Entolimod	TLR5		NK cell/neutrophil mobilization	Cancer therapeutic	^184–186^
		Mobilan Entolimod in combination with a TLR5 expressing cassette	Self-activating TLR5	Cancer therapeutic	^123^
Imidazoquinoline	TLR7/8	Imiquimod	Cytokine response	Vaccine adjuvant against HPV	^187^
		Resiquimod	T cell recruitment and expansion	T cell lymphoma therapeutic	^188^
CpG ODNs	TLR9	CpG1018	High antibody titer induction	Vaccine adjuvant against HBV (Heplisav-B)	^179^
		Class A (type D), a central phosphodiester region containing a palindromic CpG motif and 5′ and 3′poly (G) in phosphorotioate backbone	Strong pDC activator/NK stimulation	Vaccine adjuvant against viral infections	^189^
		Class B (type K), phosphorotioate backbone	B cell activator		
		Class C, CpG motif and a 3′ palindrome	Strong pDC/B cell activator		
		Class P, two palindromes in a phosphorotioate backbone	Strong pDC activator		
		IC31 ODN1a (deoxy-inosine/deoxy-cytosine) and synthetic antimicrobial peptide KLK	In vitro induction of potent Th1 and cytotoxic responses	Vaccine adjuvant against *M. tuberculosis*	^190,191^
		MGN1703 DNA molecule in a dumbbell shaped structure and non-methylated CG sequences at each end	Immunostimulatory activity	Cancer therapeutic	^192^

**Fig. 4:**
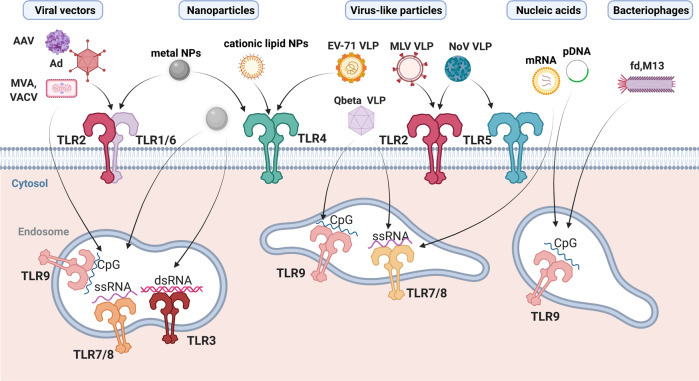
TLR sensing of viral patterns within vaccines. Viral motifs such as Env proteins, RNA, or DNA of AAV, Ad, and vaccinia viral vectors can bind and trigger the cell surface TLR2 and the endosomal TLR3, TLR7, TLR8, and TLR9 sensors, while specific structures of VLP capsids can be sensed by TLR2, TLR4, and TLR5, as described for NoV, MLV, and enteroviruses. In some cases, the self-packaging of nucleic acids during VLP assembly can lead to TLR7/8 and TLR9 activation. Metal NPs can be sensed by TLR3, TLR4, TLR6, and TLR7, depending on their composition, while lipid-based NPs can be recognized via TLR2 heterodimers. Nucleic acid-based vaccines (i.e., naked plasmid DNA or liposome-encapsulated mRNA vaccines) trigger TLR7/8 or TLR9 pathways. As natural nanoparticles, bacteriophages are internalized by immune cells and can reach endosomal compartments, where their nucleic acids are sensed by intracellular TLRs. Filamentous phages, like M13 and fd, can activate TLR9 via CpG-rich DNA sequences. Created with BioRender.com.

The increased knowledge regarding the expression and function of TLRs and their roles in response to viral infections might guide the development of vaccine platforms bearing intrinsic TLR PAMPs and/or formulated with TLR agonists for eliciting effective and long-lasting immune responses (Fig. 4).

### Nucleic acid-based vaccines

Nucleic acid-based vaccines represent a promising, effective and versatile strategy to face emerging infectious diseases, as recently outlined by the encouraging results from mRNA-based vaccines against SARS-CoV-2^105^. mRNA-based vaccines represent a case of endogenous adjuvanticity since the mRNA molecules are strong activators of TLR7/8. Additionally, these platforms could be easily manipulated to tune the immunostimulatory activity^106^. RNActive® vaccines, for instance, have been formulated in combination with the protamine cationic peptide and reported to be able to activate TLR7^107^, while the introduction of chemically modified nucleosides (pseudouridine or 1-methylpseudouridine) can reduce type I IFN production, thus downmodulating the immunostimulatory effect^108^.

DNA vaccines, based on a plasmid DNA encoding one or more antigens, are endowed with a built-in adjuvant: the unmethylated CpG motifs responsible for TLR9 activation. Thus, they can induce innate immune responses, as previously reported^109,110^. However, an innate immune response has also been described in TLR9^−/−^ mice, likely due to the role of a non-canonical IkB kinase^111^. To date, DNA-based vaccines have been licensed only for veterinary use^112^, and several attempts to improve the efficacy of these vaccines have been described, including the incorporation of a synthetic CpG cassette as TLR9 agonist^113^.

### Viral vectors

Several viruses are employed as vaccine vectors, with the most advanced platforms based on adenoviruses, adeno-associated viruses (AAVs), and poxviruses. AAVs are largely used both as vaccine carriers and for gene delivery. Although the induction of immunity toward the delivered target is desirable in case of a vaccine, the anti-vector immunity could reduce the vaccine efficacy. AAVs induce activation of innate immune responses in pDCs via the sensing of the viral genome by TLR7/TLR9, leading to high production of type I IFNs^114,115^. Type I IFNs produced by pDCs activate conventional DCs in trans, allowing optimal cross-presentation of AAV capsid antigens and subsequently CD8^+^ T cell responses^116^. In addition, in the liver, human non-parenchymal cells have been shown to be able to sense AAV vector capsid motifs at the cell surface through TLR2, leading to the production of TNF-α, IL‐6, and IL‐8^117^. Similarly, adenoviral vectors (AdV) induce innate immune responses through TLR2 and TLR9. The adenoviral DNA is sensed by TLR9 that promotes innate immune responses by triggering MyD88 intracellular signaling pathways characterized by high release of type I INFs in pDCs^118^. TLR2 activation by AdV capsid motifs can influence the induction of neutralizing antibodies against the virus itself, as well as against the delivered transgene^119^. Conversely, TRIF-dependent TLR4 signaling can act as a negative regulator of AdV-induced innate immune response^120^.

Poxviral vectors have been shown to activate both TLR-dependent and TLR-independent pathways. For instance, modified Vaccinia virus Ankara activates MyD88 signaling, probably via TLR9^121^, while VACV activates MyD88 cascade via TLR2, likely through Env or the core proteins^118^.

Several strategies have been applied to modulate immunogenicity of viral vectors, i.e., through co-expression of TRAM along with the transgene^122^, or by introducing a self-activating TLR5 signaling cassette^123^.

### Bacteriophages

Bacteriophages are viable platforms broadly utilized both for therapeutic strategies and for antigen display. *Lactobacillus, Escherichia*, and *Bacteroides* bacteriophages have been shown to stimulate a strong IFN-γ production by CD4^+^ T cells in germ-free mice. The IFN-γ production is mediated by DCs sensing the phage genome^124^. DCs exposed to bacteriophage or phage DNA can produce additional cytokines, like IL-12, IL-6, IL-10, and IFN-γ-induced protein 10, that induce IFN-γ production by T cells.

Bacteriophages can enter into eukaryotic cells by different mechanisms, including endocytosis, transcytosis, opsonization, and complement-mediated internalization, or after phagocytosis of phage-infected bacteria^125^. Upon entry, bacteriophages can remain in cytosol or enter the lysosomal pathway. Degradation of the viral capsid allows the release of phage nucleotides that can be sensed by TLRs, including TLR3, TLR7/8, and TLR9, thus activating the immune responses^126^.

Filamentous bacteriophages possess a ssDNA genome containing CpG sequences and can interact with TLR9–MyD88–NF-κB axis, shaping immune responses^127^. Thus, they have been mainly proposed for innovative vaccine formulations against cancer and infectious diseases^128–130^. Tumor-specific M13 filamentous bacteriophages, developed to target tumor microenvironment, can activate macrophages and tumor-associated macrophages, inducing secretion of pro-inflammatory mediators via MyD88. These inflammatory factors can recruit neutrophils and potentiate their antitumor cytotoxicity, mediating tumor destruction^131^. Similarly, filamentous bacteriophages *fd*, a phage infecting *Escherichia coli* cells, engineered to express human protozoan *Trypanosoma cruzi* peptides and used in a mouse model of *T. cruzi* infection, showed the induction of both humoral and cytotoxic protective responses, through a TLR9-dependent mechanism^132^. The active targeting of filamentous bacteriophage into DC endolysosomal compartments can further boost innate and adaptive immune responses evoked by bacteriophage administration through upregulation of MyD88 and subsequent DC maturation and cytokine production^133,134^.

M13 bacteriophage induces high levels of IgG2b, IgG2c, and IgG3 antibodies after priming, and IgG2b and IgG1 after boosting. The antibody response was totally abrogated in MyD88-deficient mice and reduced in TLR2^−/−^, TLR4^−/−^, and TLR7^−/−^ mice. Surprisingly, TLR9^−/−^ mice instead produced high titers of IgG1 antibodies after priming, while IgG2b titers resulted higher compared to wild-type mice. These findings suggest a role of TLR9 in the magnitude of antibody response and in the regulation of the isotype switching^135^. Moreover, bacteriophages depleted of their genome can exert some MyD88-mediated anti-inflammatory effects resulting from an interaction between TLRs and motifs contained in the phage proteins or coat derived-peptides^136^.

### Virus-like particles (VLPs)

The study of viral characteristics, including shape and size, has prompted the field of nanotechnologies to develop VLPs. VLPs are scaffolds based on one or more viral structural proteins, capable of self-assembling in supramolecular structures with the same or similar structure as the native virions. They do not contain genetic material and thus are unable to replicate, recombine, or revert to virulent strains, showing a high safety profile compared to traditional vaccines based on inactivated or attenuated viruses^137^. Due to their safety profile and ability to harbor viral epitopes in their native structure, VLPs are widely used as vaccine platforms^138,139^.

Mimicking the viral structure, VLPs also retain many motifs that could be recognized by PRRs expressed by antigen-presenting cells, thus stimulating innate and adaptive immune responses^140^. It is therefore not surprising that some VLPs are immunogenic by themselves. As they lack genetic material, activation of TLRs mediated by VLPs is not related to the NA sensing but rather to receptors that recognize specific structures of the viral capsids. EV71-based VLP, developed as a vaccine against seasonal epidemics of hand-foot-and-mouth disease, was able to trigger innate immune responses activating TLR4. Moreover, anti-TLR4 antibodies partially inhibited EV71 VLP binding to DCs, suggesting that this receptor is used by the virus to enter into cells^141^. Norovirus (NoV) VLPs, developed against the NoV GII.4 strain responsible of non-bacterial gastroenteritis, have been shown to directly interact with TLR2 and TLR5 with their subsequent activation, suggesting that these TLRs may be responsible for the immune responses to NoV^142^.

The TLR pathway is also activated by MLV-derived VLPs. Indeed, BMDC activation was reduced in MyD88^−/−^ DCs exposed to these VLPs. Upregulation of MyD88 and TLR2 gene expression in DCs confirmed that the VLP immunogenicity was TLR2 mediated^143^. In contrast to native viruses, VLPs do not contain NA-derived PAMPs that can trigger NA-sensing TLRs. However, the VLP immunogenicity might be enhanced by attaching or incorporating in vitro adjuvant sequences into its structure^144,145^.

For instance, filovirus VLP adjuvanted with different TLR agonists (poly-ICLC, MPLA, CpG ODN2395, and allydrogel) showed enhanced long-term protection against the Ebola virus, with the poly-ICLC being the most effective adjuvant in eliciting strong and Th1-skewed antibody responses^146^. The incorporation of non-coding ssRNA sequences by the VLPs can trigger TLR7/8, improving DC activation and priming CD8^+^ and CD4^+^ T cell responses. Transcriptomic analysis of retrovirus-based VLP-exposed DCs showed positive regulation of TLR signaling pathways and revealed that non-coding RNA encapsulation is able to induce increased expression of *Irf1* and other IFN-related genes such as *Mx2*, *Oas1*, *Oas2*, *Socs3*, and *Irf7* genes, which are associated with Th1-biased responses^143^.

The type of NA packaged in the VLPs can modulate the outcome of the immune response. For instance, different kinds of RNA (prokaryotic RNA, eukaryotic RNA, and transfer RNA) packaged into VLPs induce different IgG isotypes. Eukaryotic RNA incorporation preferentially orients the IgG response to the IgG1 subclass, while prokaryotic RNA induces switching toward the IgG2 isotype. Moreover, IFN-α produced by pDC-sensing RNAs induces in B cells the upregulation of TLR7 and MyD88, required for the isotype switching^147^. Similarly, Qbeta-based VLPs, packaging distinct classes of NAs that are ligands for TLR7 and TLR9, showed distinct transcriptional signature and cytokine production in DCs^148^.

### Synthetic nanoparticles (NPs)

With the emergence of modern nanotechnology, several nanometric delivery systems have been developed for the formulation of protein- or NA-based vaccines. NPs delivering antigens have been adjuvanted with different TLR agonists, including CpG ODN^149^, polyI:C^150^, Imiquimod^151^, PAM_3_CSK_4_^152^, and MPL^153^. Lipidic NPs^154^, metal-based nanostructures^155^, and biodegradable polymeric nanomaterials^156^ were employed for efficiently delivering therapeutics together with TLR ligands to stimulate cell surface or intracellular TLRs or to limit potential side effects due to systemic administration of free synthetic adjuvants^157^. TLR ligand-adjuvanted NPs are usually formulated as solid particles with a size ranging from 50 to 500 nm, with antigen and adjuvant entrapped or adsorbed on the surface of the particles. Indeed, immunization with TLR7/8 or TLR9 ligands and ovalbumin (OVA)-encapsulating poly(lactic-co-glycolic) acid (PLGA) NPs induces superior humoral and cellular immune responses with local immune activation, but attenuated systemic inflammation, compared to free TLR agonists and OVA-PLGA administration^158^.

Recently, SARS-CoV-2 S receptor-binding domain recombinant protein-encapsulated NPs were adjuvanted with different adjuvants, including AS37, a TLR7 agonist adsorbed to Alum, and CpG 1018-Alum. All the formulations induce the production of neutralizing antibodies and CD4^+^ T cells, conferring protective immunity against SARS-CoV-2 infection in non-human primates^159^.

Although most TLR ligands stimulate a Th1-skewed response, the use of TLR agonists with structural difference or in combination with other non-TLR adjuvants can help to shape desired immune responses^160^. A novel approach using dual TLR agonists for an efficient nanoparticulate adjuvant-vaccine formulation was recently developed. The TLR7/8 agonist resiquimod (R848) or the TLR4 agonist MPLA were encapsulated in PLGA conjugated to polyethylenimine (PEI) and co-assembled with the TLR9 agonist CpG ODN to form a tripartite formulation with two TLR agonists located inside and outside NPs and PLGA/PEI NPs as a delivery system for the model antigen OVA. PLGA NPs containing dual TLR agonists were assayed in vivo and were able to induce stronger responses compared to single adjuvanted OVA NPs. In addition, a Th1-skewed cytokine (IFN-γ) and antibody (IgG2a)-mediated responses were achieved after in vivo administration of TLR9 and TLR7/8 adjuvanted formulations. In contrast, the highest level of IgG1, corresponding to Th2-oriented response, was obtained using the dual TLR4 and TLR9 agonists, suggesting how mixing adjuvants for different TLRs can modulate the ratio of Th1/Th2 immune responses^161^.

Although often considered inert, some types of NPs can be sensed by innate immunity receptors, modulating the adaptive responses. Metal oxide NPs are promising nanosystems with a wide range of applications in nanomedicine, including biomedical imaging, drug and gene delivery, biosensing, and antimicrobial treatments. Fe_3_O_4_, TiO_2_, ZnO, CuO, Ag_2_O, and AlOOH NPs have been recently demonstrated to promote upregulation of TLR4 and TLR6 expression in macrophages, inducing cytokine synthesis and pro-inflammatory responses^162,163^, while ZrO_2_ and TO_2_ NPs increased the expression levels of TLR3 and TLR7^164^. Poly(gamma-glutamic acid) NPs induce DC maturation via TLR4–NF-κB pathway activation^165^, while cationic lipid nanocarriers, such as polyamine lipids bearing C14-acyl chains, have recently been shown to induce TLR2 dimerization with both TLR1 and TLR6, in a similar manner to the Pam_3_CSK_4_ synthetic lipopeptide^166^.

## Concluding remarks

TLRs play a pivotal role in sensing pathogens. Upon viral infection, activated TLRs trigger downstream signaling pathways that culminate in the host antiviral immune responses. On the other hand, in a constant effort to escape from immunosurveillance, viruses have developed several mechanisms to counteract the host controls. These include interference with TLR expression, mimicking the host molecular structures, and cleavage of host receptors or mediators involved in the TLR signaling cascades. Overall, a deep understanding of viral innate sensing mediated by TLRs is currently guiding the development of innovative vaccine formulations bearing intrinsic TLR PAMPs and/or formulated with TLR agonists to fight viral infections. Here we described vaccine platforms exploiting NAs, viral vectors, bacteriophages, VLPs, and synthetic NPs. Among them, only vaccines based on VLPs, mRNA molecules, and viral vectors have been licensed for human use to date, with the latter two playing a fundamental role in fighting the spread of SARS-CoV-2 infection worldwide. Considering the recent enthusiastic results concerning the efficacy of mRNA- and viral vector-based vaccines that the global scientific community is constantly collecting during the COVID-19 pandemic, it is reasonable to foresee that in the near future vaccines employing these platforms would be used to prevent the infection of emerging/re-emerging pathogens or to face those microbes for which efficient countermeasures do not actually exist.
